# Soft Polydimethylsiloxane-Supported Lipid Bilayers for Studying T Cell Interactions

**DOI:** 10.1016/j.bpj.2020.11.021

**Published:** 2020-11-26

**Authors:** Anna H. Lippert, Ivan B. Dimov, Alexander K. Winkel, Jane Humphrey, James McColl, Kevin Y. Chen, Ana M. Santos, Edward Jenkins, Kristian Franze, Simon J. Davis, David Klenerman

**Affiliations:** 1 Department of Chemistry, University of Cambridge, Cambridge, United Kingdom; 2 Department of Physiology, Development and Neuroscience, University of Cambridge, Cambridge, United Kingdom; 3 Radcliffe Department of Medicine and MRC Human Immunology Unit, John Radcliffe Hospital, University of Oxford, Oxford, United Kingdom

## Abstract

Much of what we know about the early stages of T cell activation has been obtained from studies of T cells interacting with glass-supported lipid bilayers that favor imaging but are orders of magnitude stiffer than typical cells. We developed a method for attaching lipid bilayers to polydimethylsiloxane polymer supports, producing “soft bilayers” with physiological levels of mechanical resistance (Young’s modulus of 4 kPa). Comparisons of T cell behavior on soft and glass-supported bilayers revealed that whereas late stages of T cell activation are thought to be substrate-stiffness dependent, early calcium signaling was unaffected by substrate rigidity, implying that early steps in T cell receptor triggering are not mechanosensitive. The exclusion of large receptor-type phosphatases was observed on the soft bilayers, however, even though it is yet to be demonstrated at authentic cell-cell contacts. This work sets the stage for an imaging-based exploration of receptor signaling under conditions closely mimicking physiological cell-cell contact.

## Significance

Because T cell activation is guided by signaling proteins functioning at cell-cell contacts but the imaging of proteins inside such contacts is not straightforward, studies of T cell interactions usually exploit model systems reflecting either the stiffness of cellular substrates or the fluidity of proteins and lipids, e.g., using glass-supported lipid bilayers. We present a simple method for forming bilayers on substrates of controlled stiffness as low as 4 kPa. The method allowed us to image the diffusion and distribution of important signaling proteins and to study the early stages of T cell signaling in a setting approximating physiological stiffness.

## Introduction

Like other receptors such as growth factor and G-protein-coupled receptors, individual T cell receptors (TCRs) appear to initiate downstream signaling after binding their ligands (1). But unlike these other receptors, the ligands of the TCR are exclusively cell-bound. To understand how T cell activation is initiated, therefore, it will be necessary to study the interfaces of interacting cells. For now, the best way to study cell surface phenomena is to use total internal reflection fluorescence (TIRF)-based imaging of model cell surfaces. TIRF imaging of glass-supported lipid bilayers (SLBs) used as model antigen-presenting cell (APC) surfaces, a method pioneered by Dustin and collaborators (2), has transformed our understanding of T cell activation (3). A special advantage of the bilayer approach is that the bilayers can be easily and systematically functionalized by attaching the soluble extracellular regions of receptor ligands and other components of the membrane, allowing quantitative analysis of T cell responses. In recent years, however, it has become apparent that T cell behavior is also affected by the mechanical properties of surfaces they encounter (4, 5, 6, 7, 8, 9), and glass is 10^7^- to 10^8^-fold stiffer than eukaryotic cells (10).

Cells and tissues exhibit a wide range of stiffnesses, from ∼100 Pa to 8 kPa for lymphocytes up to gigapascals for bone (10). This suggests that many cells will have to be mechanosensitive to correctly navigate and/or respond to their local environments. It might have been expected that lymphocytes would constitute a special case of mechanosensitive cells because they traverse great distances across multiple organs and tissues in the course of interrogating other cells for signs of infection and malaise. Accordingly, multiple groups have shown that lymphocytes, i.e., both T cells (4,6) and B cells (11,12), are affected by surface mechanical properties. For example, T cells interacting with elastomer micropillar arrays undergo cytoskeletal changes and intracellular signaling responses that vary with pillar length and flexibility (13). Substrate stiffness also modulates T cell migration and morphology and alters the expression of activation-induced immune-system-, metabolism-, and cell-cycle-related genes (4,8).

Consistent with such observations, a considerable body of data implies that T cells use force-sensing mechanisms acting through the TCR to initiate signaling (14, 15, 16, 17). Paradoxically, however, T cells are unusually soft (∼85 Pa (10)), implying that membrane deformability may also be important. One explanation for TCR triggering that incorporates this feature of the membrane proposes that signaling relies on the passive exclusion of large receptor-type tyrosine phosphatases, such as CD45, from small regions of contact with APCs where TCRs engage their ligands (18,19). Local removal of phosphatase activity in this way is suggested to favor receptor phosphorylation by nonexcluded kinases attached to the inner leaflet of the T cell membrane. According to this idea, the formation of small contacts—aided by membrane deformability—is critical for ensuring that T cell responses are antigen dependent (20,21). CD45 has been found to exit from micrometer-sized regions of T cell contact with glass surfaces and bilayers (20, 21, 22), but when live T cell and APC contacts were imaged using lattice light sheets, CD45 exclusion was not observed (23).

Here, we establish a new, to our knowledge, method for attaching bilayers to soft polydimethylsiloxane (PDMS) supports. Using the new, to our knowledge, method and fluorescence imaging, we probe the stiffness dependence of membrane protein reorganization and receptor-proximal signaling as T cells form contacts with apposing, cell-like surfaces.

## Materials and Methods

### Key resources

See Table 1 for resources used for this work.

**Table 1:** Key Resources

Reagent or Resource	Source	Identifier
**Antibodies**		

Anti-CD3 Ab	antibody purification service at the Human Immunology Unit, WIMM, University of Oxford	OKT3
Anti-CD45 Fab	GAP8.3 hybridoma was obtained from ATCC (HB-12; Manassas, VA)	Gap 8.3
Anti-CD3 Fab	UCHT1 hybridoma was a generous gift from Dr. Neil Barclay, Sir William Dunn School of Pathology, University of Oxford	UCHT-1

**Chemicals, Peptides, and Recombinant Proteins**		

Fluo-4 AM	Thermo Fisher Scientific	F14201
CaCl2	Thermo Fisher Scientific	C|1400|53
rCD2	(20)	
pMHC (HLA-A) 9V	(28,54)	
POPC	Avanti Polar Lipids	850457C
DGS-NTA(Ni) (nickel salt)	Avanti Polar Lipids	790404C
Oregon Green-DHPE	Thermo Fisher Scientific	O12650
SYLGARD 184	Sigma-Aldrich	761036
NuSil GEL-8100 2pint kit	Polymer Systens Technology	GEL-810
Alexa488 NHS ester	Invitrogen, Thermo Fisher Scientific	A20000
Alexa647 NHS ester	Invitrogen, Thermo Fisher Scientific	A2006

**Critical Commercial Assays**		

Pierce Fab Preparation Kit	Thermo Fisher Scientific	44985

**Experimental Models: Cell Lines**		

Jurkat rCD48	(20)	
Jurkat J8 GCaMP	(54)	

**Software and Algorithms**		

MATLAB	The MathWorks	2019
ImageJ	National Institutes of Health	1.52
OriginPro	OriginLab	2020
SymphoTime 64	PicoQuant	2.4.4874

**Other**		

SHOCON-10 cantilever	AppNano	SHOCON-10
Polystyrene beads	microParticles	PS-R-37.0
Arrow TL1 cantilevers	NanoWorld	Arrow TL1
Micro Bio-Spin 6 columns, Tris buffer, 25	Bio-Rad (Hercules, CA)	7326221
Cover glasses	VWR International (Lutterworth, UK)	size 1
CultureWell, chambered cover glass	Grace Bio-Labs	CWCS-50R-1.0, 103350
Syringe filter, 20 nm	Whatman	Anotop 25, 6809-2002

### PDMS fabrication

For the fabrication of 4 kPa and 1 MPa gels, two commercially available polymer reagents were mixed as follows: SYLGARD184 (Sigma-Aldrich, St. Louis, MO) was mixed in a 1:10 ratio (i.e., curing agent/monomer ratio) to prepare 1 MPa gels. NuSil Gel 8100 (Polymer Systems Technology, High Wycombe, UK) was prepared following the manufacturer’s instructions (1:1 A and B components). The gel mix was then supplemented with 1% of the prepared Sylgard184 mix. After gentle stirring, the gels were spread on glass slides using razor blades, with 0.0625 mm Tape (3M 810 TAPE Scotch Magic Tape) as spacer, and subsequently cured at 65°C for 13 h, after which they were ready for experiments. The Young’s modulus of the soft PDMS gels was found to be 4.59 ± 0.35 kPa (n = 16), and creep response experiments showed a low viscous contribution, indicating that the gels were mostly elastic in their response (see Fig. S1).

### PDMS stiffness measurements

All PDMS substrate stiffnesses were measured using either a JPK CellHesion 200 or JPK NanoWizard 3 atomic force microscope (Bruker, Billerica, MA) operated in force spectroscopy mode and Arrow TL1 cantilevers (NanoWorld, Neuchâtel, Switzerland) onto which a polystyrene bead (PS-R-37.0; microParticles, Berlin, Germany) had been glued. PDMS stiffnesses were measured in phosphate-buffered saline (PBS), with 250 *μ*g/ml bovine serum albumin to prevent tip and sample adhesion. Recorded indentations were processed using the JPK data processing software to extract the Young’s modulus from the Hertz model (24). Force spectroscopy was also used to detect bilayer push-through events using SHOCON-10 cantilevers (AppNano, Mountain View, CA). These were then analyzed using a purpose-written script in MATLAB (The MathWorks, Natick, MA).

### SUV preparation

Small unilamellar vesicle (SUV) solutions were prepared in glass vials where the amount of desired lipid was added from stock solutions using glass syringes. After drying the lipids under nitrogen flow and at least 1 h inside a desiccator, PBS was added to the vial to yield 10 mg/mL SUV stock solutions. After a short vortexing step, the solution was sonicated in a water bath until the solution became clear. Bilayers were prepared from SUV solutions containing POPC (1-palmitoyl-2-oleoyl-*sn*-glycero-3-phosphocholine, 850457C; Avanti Polar Lipids, Alabaster, AL) and 0.01% Oregon Green-DHPE (1,2-dihexadecanoyl-*sn*-glycero-3-phosphoethanolamine, O12650; Thermo Fisher Scientific, Waltham, MA) for the lipid diffusion experiments. To functionalize the bilayers with proteins, SUV solutions of POPC with DGS-NTA(Ni) (1,2-dioleoyl-*sn*-glycero-3-[(N-(5-amino1-carboxypentyl)iminodiacetic acid)succinyl] (nickel salt), 790404C; Avanti Polar Lipids) at 2% DGS-NTA(Ni), 98% POPC were prepared.

### Bilayer preparation on PDMS and glass

After the curing of the PDMS, commercial culture chambers (CWCS-50R-1.0 103350; Grace Bio-Labs, Bend, OR) were carefully peeled off their glass supports and placed on the PDMS gels to form sample chambers. Before all experiments, cells were placed in one sample chamber to ensure that the PDMS surface would be within the working distance of the objective used. To prepare the bilayers, each remaining well was filled with 10 *μ*L of 1 mM CaCl_2_ solution, which was filtered through a 20 nm filter (Anotop 25, 6809-2002; Whatman, Maidstone, UK). The gels were then incubated overnight at 4°C in an improvised humidity chamber formed by a water bath placed in a glass petri dish that was sealed with parafilm. After the incubation, the slides were washed three times with PBS, and then SUV solution was added at 1 mg/mL and additional calcium added at a final concentration of 10 *μ*M. After 30 min incubation and five washing steps with PBS to remove free SUVs and calcium, proteins were added and incubated for 60 min. The proteins bound the Ni^+^ chelating NTA lipid via 2× hexahistidine tags. Before experiments, the bilayers were washed five times with PBS to remove unbound proteins. Bilayers were either functionalized with 25 nM rCD2-Alexa647 (∼100–200 mol/*μ*m^2^) alone or with 70 nM pMHC (∼600–800 mol/*μ*m^2^). Bilayers were checked for mobility before each experiment.

Bilayers on glass were prepared on Piranha-cleaned glass slides followed by a 30 min arrgon plasma treatment (Hatrick Plasma) before adding the SUV solution to the cleaned glass. After 30 min to allow bilayer formation on the glass, samples were washed three times with PBS.

### Cell culture

The Jurkat rCD48 cell line was generated via lentiviral transfections with a pHR lentiviral vector as described previously (20,21). The expression of rCD48 matched physiological CD2 levels at ∼30,000 molecules per cell. The J8 Jurkat line was engineered to express the calcium indicator GCaMP7s (25,26), as well as the NY-ESO/HLA-A2 reactive 1G4 TCR (27). For expressing the 1G4 TCR, the endogenous TCR genes were inactivated in Jurkat cells using CRISPR and replaced with cDNA encoding the wild-type 1G4 TCR, expressed at physiological levels (∼30,000/cell) via retroviral transduction. The gene encoding CD4 was also disrupted and genes expressing CD8*αβ* used in its place. All T cells were cultured in phenol-red-free RPMI supplemented with 10% fetal calf serum, 1% HEPES buffer, 1% sodium pyruvate, and 1% penicillin-streptomycin.

### Proteins and labeling

rCD2 (residues 23–219, P08921; UniProt) was cloned into the pHR vector with a 6H-SRAWRHPQFGG-6H tag at the C-terminus. rCD2 was expressed by lentiviral transduction using HEK 293T cells and purified using Ni-NTA beads and size-exclusion chromatography. Soluble pMHC (HLA-A2 complexed with the 9V variant of NY-ESO) was produced as previously described (28). A fragment antigen binding (Fab) reactive with human CD45 was produced by digestion of Gap8.3 antibody. rCD2, UCHT-1 Fab, and Gap8.3 Fab were labeled with Alexa 647 and 488 using the succinimidyl ester method as needed. For bulk imaging and calcium signaling experiments, cells were labeled with Gap8.3 Fab at 10 *μ*g/mL and 5 mM Fluo-4 for 15 min in phenol-red-free RPMI at 37°C and subsequently washed three times in PBS. To perform single-molecule tracking experiments, cells were labeled with Gap8.3 Fabs at 1 nM for 15 min and then washed three times in PBS.

### Imaging

#### FRAP

The mobile population in the bilayer was assessed using fluorescence recovery after photobleaching (FRAP). In these experiments, 10 frames were recorded before bleaching performed by removing beam expanders. Postbleaching, the intensity was recorded at 1 s intervals over 3 min using a 100× 1.49 NA Nikon TIRF objective (Nikon, Tokyo, Japan).

#### TIRF

TIRF imaging was performed at 37°C or room temperature when indicated using a 100× 1.49 NA Nikon TIRF objective and 488 nm (Spectra-Physics-488, 100 mW; Spectra-Physics, Santa Clara, CA) and 638 nm (Cobolt 06-MDL 638 nm 180 mW; Stockholm, Sweden) lasers on a custom-built TIRF setup. The fluorescence signal was either split using a DualView2 (Teledyne Photometrics, Tucson, AZ) or passed through an OptoSpin spinning filter wheel (Cairn Research, Faversham, UK; filter: Alexa 488—FF01-525/50-25, Semrock, Rochester, NY; Alexa 647—FF02-632/22-25, Semrock) before being recorded with an iXon Andor EMCCD camera (Andor, Belfast, UK). Image stacks were acquired at exposure times of 10–30 ms. When imaging with labeled Fabs, imaging was performed at room temperature within 15 min because of the high off rates of the Fabs.

#### Calcium imaging

Calcium imaging was performed in epi-illumination mode at 37°C using a 20× 0.5 NA Plan Fluor Nikon objective with frames captured every second at 30 ms exposure for 10 min. Before experiments, cells were placed onto the surfaces and imaged on OKT3-coated glass (10 min coating with 10 *μ*g/mL OKT3, three times washed with PBS) to confirm the responsiveness of the cells. The fraction of cells triggering on OKT3 was generally found to be 60–80%.

#### FCS

All fluorescence correlation spectroscopy (FCS) data were acquired on a MicroTime 200 confocal setup (PicoQuant, Berlin, Germany) equipped with a HydraHarp 400 (PicoQuant) and hybrid PMTs (PMA Hybrid 40; PicoQuant). The experiments were carried out using a pulsed picosecond laser diode (LDH-DC-485; PicoQuant) and a UplanSApo 60× 1.2 NA water objective (Olympus, Tokyo, Japan) (dichroic: 405 + 485, emission filter: BLP01-488R-25). The confocal volume was calibrated using rhodamine 6G solution at 10 nM. The analysis was performed using the SymPhoTime 64 software (PicoQuant), which generated and fitted the autocorrelation curve G(t) to an in-house-built two-dimensional diffusion extended triplet equation.

### Image analysis

#### FRAP

The mobile fraction and diffusion coefficient were calculated as follows. After background subtraction and Gaussian smoothing, the first frame after bleaching was normalized to the frame before bleaching. The effective beam radius (w) was obtained by fitting to the exponential of a two-dimensional Gaussian function, as described in (29). The intensity trace was then normalized using a reference region, to correct for photobleaching. The mobile fraction (*f*_*mobile*_) was obtained from

$$f_{mobile}=\frac{F_{\infty }-F_{0}}{F_{i}-F_{0}},$$

where *F*_*i*_ is the intensity before bleaching, *F*_0_ the intensity immediately after bleaching, and *F*_∞_ is the intensity at the asymptote. The characteristic diffusion time *τ*_*D*_ was extracted using Axelrod’s model (29,30)

$$F\left(t\right)=\sum_{n=0}^{\infty }\frac{\left(-K^{n}\right)}{n!\left(1+n\left(1+\frac{2t}{\tau }\right)\right)}\;M_{f}+\left(1-M_{f}\right)F_{0\;}$$

and used to calculate the diffusion coefficient (*D*):

$$D=\frac{w^{2}}{4\tau_{D}}$$

#### Particle tracking and diffusion analysis

Single-particle tracking and diffusion analysis was performed using a custom-written MATLAB code (31). Here, lipid diffusion in the bilayers was calculated by fitting the first five points (50 ms) of the ensemble mean-square displacement (MSD) curve to (32)

$$MSD=4Dt+4\sigma^{2}-4/3Dt$$

Jump-distance (JD) analysis was performed to fit two diffusing populations on tracks gathered from each cell. The probability distribution *P*(*r*^2^, *Δt*) of the squared distance traveled, *r*^2^, in one time step *Δt* was fitted to (31)

$$P\left(r^{2},\Delta t\right)=\;\sum_{j=1}^{n}\frac{f_{j}}{4D_{j}\Delta t}e^{-\frac{r^{2}}{4D_{j}\Delta t}}$$

For all diffusion measurements, only tracks longer than five frames were used.

#### Calcium analysis

Calcium data were analyzed using custom-written software in MATLAB. In this software, cells were identified and tracked to gather intensity traces. Peaks were then detected using the FindPeaks built-in MATLAB function. In each experiment, the number of cells exhibiting a calcium response (peaks ≥ 1) and the fraction of triggering cells that exhibited multiple peaks (peaks > 1) were calculated, along with the average time to trigger the first calcium peak, integrated peak intensity, and peak height in multiples of the baseline. The source code for the calcium analysis has been deposited in GitHub under https://github.com/janehumphrey/calcium.

#### Exclusion analysis

rCD2 and CD45 image stacks were averaged and background subtracted using the rolling background function in ImageJ (pixel size = 50). The images were then analyzed using custom-written MATLAB code in which an Otsu-generated threshold (33) was used to create a contact mask (rCD2 channel) and cell mask (contact + CD45 mask) and the mean intensity of CD45 inside $\left(\bar{CD45_{in}}\right)$ and outside of the contact $\left(\bar{CD45_{out}}\right)$ was calculated. CD45 exclusion was then set as *Exclusion* = 1 − $\frac{\bar{CD45_{in}}}{\bar{CD45_{out}}}$.

### Statistical analysis

All statistical tests were performed with OriginPro (OriginLab). For experiments for which the distribution could not be assessed, a nonparametric Mann-Whitney U test was performed to test for significant differences. Otherwise, data were tested if they followed a normal distribution using a Shapiro-Wilk test, and if the data were normally distributed, a two-sided *t*-test was performed to assess the significance of differences. If this was not the case, a Mann-Whitney U test was used.

## Results

### Creation and characterization of PDMS-supported lipid bilayers

**Figure 1:**
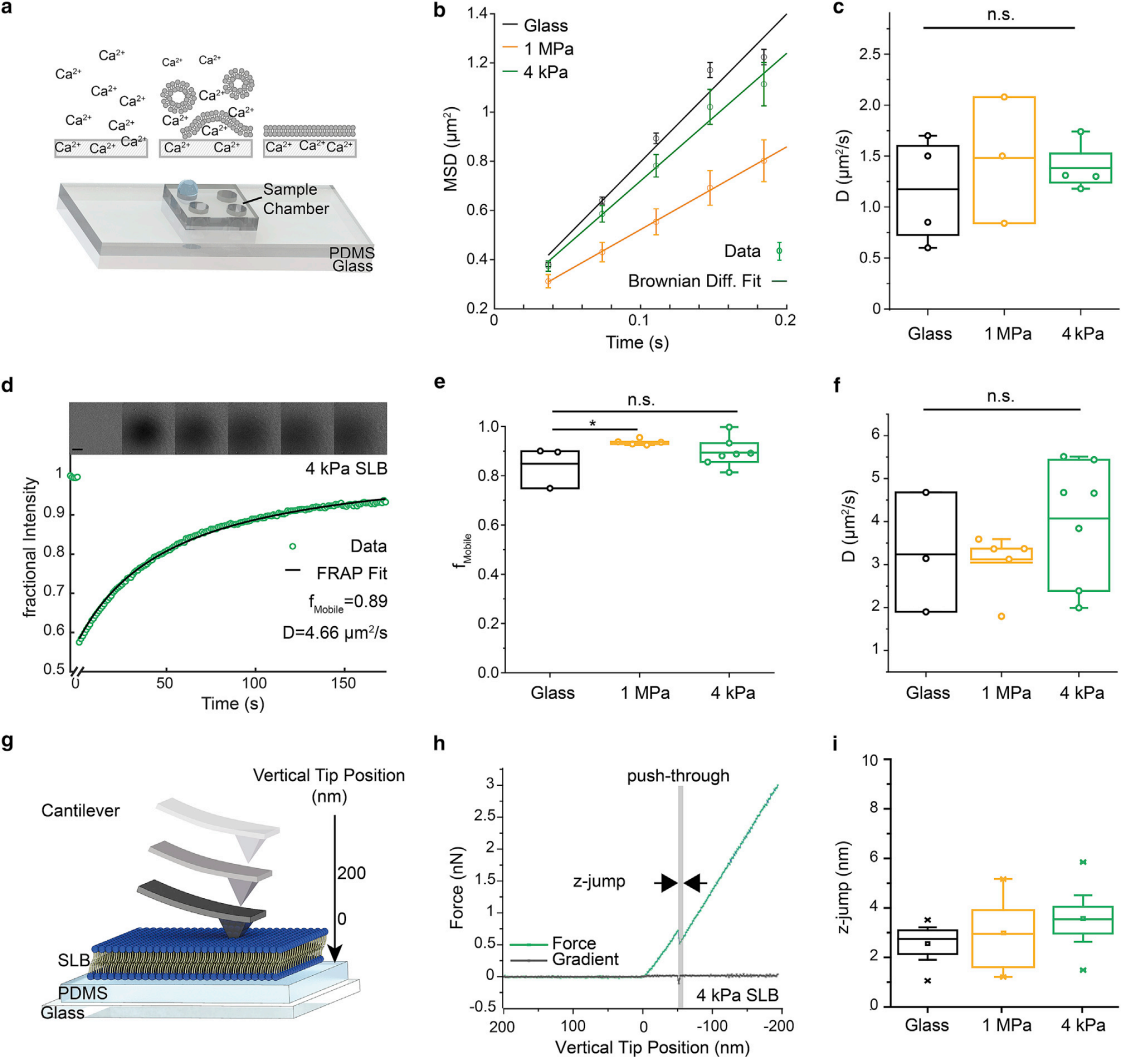
Formation and characterization of PDMS-supported bilayers. (*a*) A schematic representation of the experimental setup and the bilayer formation process is shown. (*b* and *c*) Single-molecule diffusion analysis of lipids in the PDMS-supported bilayers is given. (*b*) Representative MSD plots and fits of data acquired on SLBs formed on 4 kPa (D = 1.3 *μ*m^2^/s, *σ* = 0.116 *μ*m^2^, n_Tracks_ = 264) and 1 MPa PDMS gels (D = 0.8 *μ*m^2^/s, *σ* = 0.103 *μ*m^2^, n_Tracks_ = 452) and glass (D = 1.5 *μ*m^2^/s, *σ* = 0.117 *μ*m^2^, n_Tracks_ = 5430) are shown. Error bars indicate SEM. (*c*) Comparison of the diffusion coefficients of lipids on SLBs supported by glass and 1 MPa and 4 kPa PDMS gels is shown. Each data point represents one independent measurement of lipid diffusion at room temperature (RT) (n_tracks_ > 100) each performed on a separate bilayer. (*d*) Representative FRAP curve and fit from an SLB on 4 kPa PDMS gel are given, with raw data showing the fluorescence recovery after photobleaching, above. Scale bars, 10 *μ*m. (*e*) Mobile fractions *f*_mobile_ and (*f*) diffusion coefficients measured using FRAP are shown. In (*e*) and (*f*), each data point represents one independent experiment, performed at RT. (*g*–*i*) Mechanical characterization of SLBs on PDMS via AFM is shown. (*g*) A schematic representing the AFM cantilever approaching and contacting first the SLB and then the PDMS is given. (*h*) Representative force curve (*green*) on a 4 kPa PDMS gel is shown. Push-through events are detected via changes in the gradient of the force curve (*black*). (*i*) Boxplots of SLB thickness (z-jump) are given (number of force curves analyzed: 30 (glass), 31 (1 MPa PDMS), and 52 (4 kPa PDMS)) acquired on three separate SLBs per condition. Boxes indicate the 25 and 75% quartile, the horizontal line the mean, and whiskers the 1.5 IQR . The Mann-Whitney U test was used for statistical comparisons. To see this figure in color, go online.

To study T cell signaling on surfaces that had softnesses comparable with those of lymphocytes but retained the advantages of glass-supported lipid bilayers for imaging, we formed SLBs on PDMS supports. Whereas previous methods of attaching SLBs to PDMS were reliant on solvent or plasma treatments (7) that influence the stiffness of the PDMS support (34), our method is nonperturbative. The method involves an overnight incubation of PDMS-presenting slides with a 1 mM calcium chloride solution before bilayer formation using small unilamellar vesicles (Fig. 1 *a*). The bilayers thus formed can then be functionalized using histidine-tagged proteins in the manner of glass-supported bilayers. We found that lipid bilayers could be formed on PDMS supports of different stiffness, e.g., 4 kPa and 1 MPa. Atomic force microscopy (AFM) measurements (Video S1. Lipid Diffusion on 4 kPa and 1 MPa PDMS- and Glass-Supported Lipid Bilayers, Video S2. Diffusion of A488-Tagged UCHT1 Antibody Fab-Coupled TCRs in rCD2 Contacts Formed on Glass- and 4 kPa PDMS-Supported Lipid Bilayers, Video S3. rCD2 Contact Formation and Calcium Signaling on 4 kPa PDMS-Supported Lipid Bilayers at 37°C, Video S4. rCD2 Contact Formation and Calcium Signaling on Glass-Supported Lipid Bilayers at 37°C, Document S2. Article plus Supporting Material) confirmed that the PDMS gels exhibited predominantly elastic responses with minimal creep (Fig. S1 b), and that calcium treatment did not lead to an increase in gel stiffness (Fig. S1, c and d). Typically, the PDMS supports for the bilayers were ∼50 *μ*m in depth. For most of our functional experiments comparing the glass-supported and soft bilayers, PDMS gels of 4 kPa and 1 MPa stiffness were used as supports. The 4 kPa stiffness matches that of dendritic cells (2–8 kPa) (35), the most relevant surface for understanding T cell priming.

To confirm that the bilayers were fluid and mobile, the diffusion coefficients of the lipids along with their mobility were assessed using single-molecule tracking (Fig. 1, *b* and *c*; Video S1) and FRAP (Fig. 1, *d*–*f*). We found that 80–90% of the lipids in the bilayers on 4 kPa and 1 MPa PDMS and glass supports were mobile (Fig. 1 *e*), with diffusion coefficients between 2 and 5 *μ*m^2^/s (Fig. 1, *c* and *f*). FCS measurements gave similar values (Fig. S2). To establish that a single bilayer and not multiple layers had formed on the PDMS supports, we performed AFM force curve measurements, analyzing single push-through events using a pyramidal AFM cantilever (Fig. 1 *g*). In these experiments, the cantilever was first brought into contact with the bilayer and then, with increasing force, pushed through the bilayer and into contact with the underlying PDMS support. On 4 kPa PDMS-supported bilayers, 51 of 52 push-through experiments produced a single push-through event (see, e.g., Fig. 1 *h*), with just one force curve yielding two sequential push-through events. This indicated the formation of single bilayer sheets on the PDMS instead of multiple bilayers. Our glass- and 1-MPa-supported bilayers also behaved as expected (Fig. S3). The 1 MPa and 4 kPa PDMS- and glass-supported bilayers exhibited similar resistance to push-through (Fig. S3 b) and had similar depths (z-jumps; Fig. 1 *i*) that were in good agreement with the reported values of ∼3.9 nm for POPC bilayers (36,37). The ratio of the slopes before and after push-through for the 4 kPa PDMS-supported bilayers (Fig. 1 *h*; Fig. S3 b) was close to 1, indicating that the bilayer and underlying support were of similar stiffness.

Video S1. Lipid Diffusion on 4 kPa and 1 MPa PDMS- and Glass-Supported Lipid Bilayers

### Interactions of T cells with the PDMS-supported bilayers

**Figure 2:**
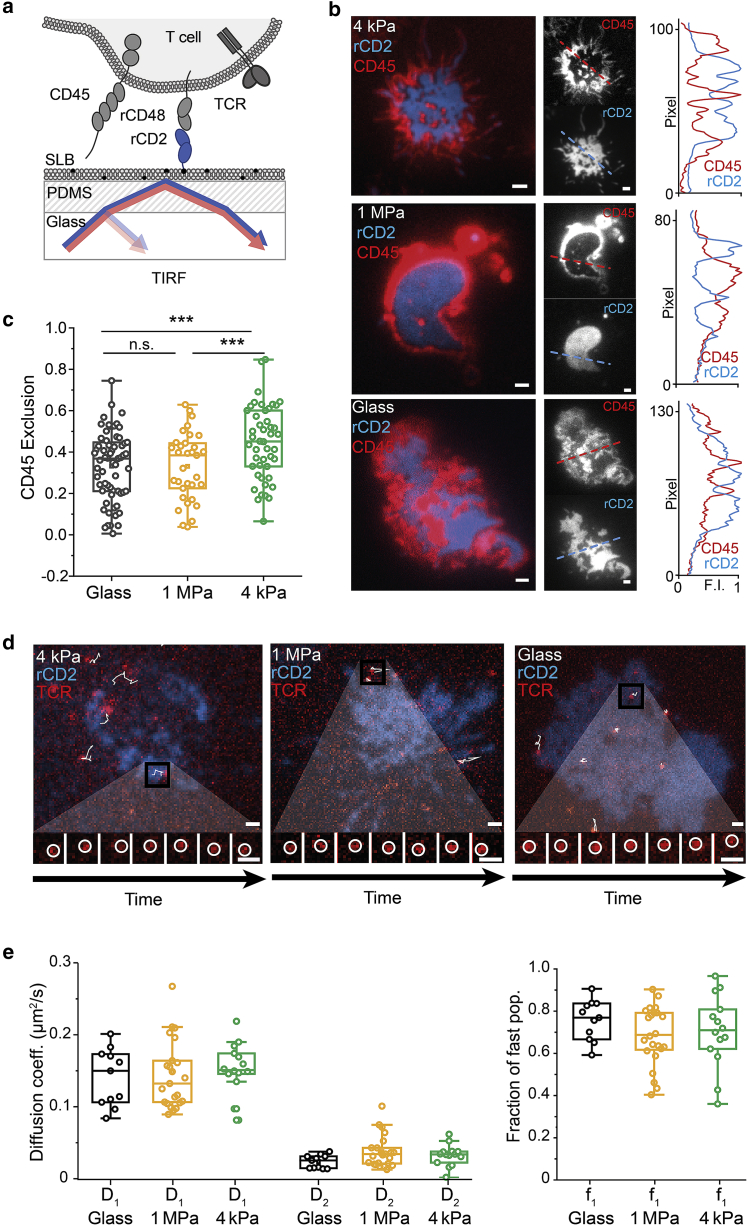
Properties of contacts with the PDMS-supported bilayers presenting the adhesion protein rCD2. (*a*) A schematic of the experiment is given. Cells expressing signaling-disabled rCD48 were deposited onto SLBs presenting rCD2. Cells were labeled with Gap8.3 anti-CD45 Fab (*b* and *c*) or UCHT1 anti-CD3*ε* Fab (*d*–*f*). (*b*) Overlay and individual TIRF images showing CD45 (*red*) and rCD2 (*blue*) fluorescence with a line profile of normalized pixel intensity on the right (*dashed lines*) are given. Scale bars, 1 *μ*m. (*c*) Boxplots of CD45 exclusion are shown. Boxes indicate the 25 and 75% quartile, the horizontal line the mean, and whiskers the 1.5 IQR. Each data point corresponds to one cell; n_cells_ = 63 (glass), n_cells_ = 33 (1 MPa), and n_cells_ = 47 (4 kPa) acquired from three independent experiments at RT. (*d* and *e*) Diffusion measurements of the TCR are shown. T cells were labeled with Alexa 488 UCHT1 Fab and deposited on rCD2 functionalized SLBs. (*d*) Shown is an overlay of single TCRs (*red*) and the rCD2 contact (*blue*) formed on 4 kPa SLB, 1 MPa SLB, and glass SLB. Local TCR tracks are overlaid (*white*), and the inset below is an example of a single TCR diffusing over time (interval 31 ms). Scale bars, 1 *μ*m. (*e* and *f*) Cell-wise two-component JD fit of the JD distributions of tracking data acquired on glass (n_cells_ = 12, n_tracks_ per cell > 30, mean track length over all cells: 20.8 ± 5.4, mean SNR: 7.8 ± 2.4), 1 MPa PDMS (n_cells_ = 24, n_tracks_ per cell > 30, mean track length over all cells: 14.3 ± 2.7, mean SNR: 6.4 ± 1.6), and 4 kPa PDMS (n_cells_ = 15, n_tracks_ per cell > 30, mean track length over all cells: 15.9 ± 4.2, mean SNR: 5.6 ± 1.6) supported bilayers obtained in two independent experiments performed at 37°C. A cell-by-cell account of SNR as well as histograms of background and localization intensities can be found in Fig. S5. (*e*) Boxplots of the diffusion coefficients of the fast diffusing population (D_1_) and the slow diffusing population (D_2_) are shown, comparing TCR diffusion acquired on glass- and 1 MPa and 4 kPa PDMS-supported bilayers. (*f*) Boxplots of the fraction of the fast diffusing population (f_1_) are given. Each data point represents a fit obtained from one cell, with boxes indicating the 25 and 75% quartile, the horizontal line the mean, and whiskers the 1.5 IQR. A two-sided *t*-test was used for statistical analysis with ^∗^*p* < 0.05, ^∗∗^*p* < 0.01, ^∗∗∗^*p* < 0.001. To see this figure in color, go online.

We used the new bilayers to examine whether size-dependent surface protein reorganization occurs at contacts of T cells with soft surfaces and the extent to which very early T cell signaling is dependent on substrate resistance, each of which is unclear. One of the advantages of PDMS as a supporting substrate is that it has a refractive index similar to glass, allowing TIRF imaging. Using this imaging mode, therefore, we set out to determine the extent to which substrate stiffness affects the nature of the contacts Jurkat T cells form with model surfaces. For this, we imaged the behavior of the large glycocalyx component and receptor-type protein tyrosine phosphatase CD45 expressed by the T cells and the small, histidine-tagged adhesion protein rat (r) cluster of differentiation antigen 2 (rCD2), inserted into the SLBs to allow for the formation of adhesive contacts (Fig. 2 *a*). CD45 was indirectly labeled with the Fab fragment of the Gap8.3 antibody (38), whereas CD2 was labeled directly on lysines with Alexa-647 dye.

We imaged Jurkat T cells expressing rCD48, the ligand of rCD2, as they formed contacts with rCD2-functionalized bilayers formed on glass and on 1 MPa and 4 kPa PDMS gels. T cells readily formed single or multifocal contacts on both the soft and stiff bilayers (Fig. 2 *b*). Cell contacts with the bilayers were marked by the accumulation of fluorescent CD2; an example of an early contact formed on a 4 kPa PDMS-supported bilayer is shown in Fig. S4. Notably, CD45 was excluded from regions of CD2 accumulation (Fig. 2, *b* and *c*; Fig. S4). Time-lapse imaging was used to follow the formation of contacts, and this showed that CD45 was excluded very early (less than 20 s; Fig. S4). Surprisingly, the extent of CD45 exclusion was slightly greater on soft versus stiff bilayers (Fig. 2 *c*). Single-molecule tracking using two-color TIRF imaging of single TCRs within the rCD2-accumulating contacts and in the absence of TCR ligands in the bilayer revealed that receptor diffusion was rapid (0.15 *μ*m^2^/s), as reported previously (39), and unaffected by bilayer stiffness (Fig. 2, *e* and *f*; Video S2). Similarly, large fractions of the TCRs were mobile in both types of bilayers (Fig. 2 *f*). Here, the PDMS did not affect the background during acquisition, but it did reduce the signal intensity on 4 kPa gels, which resulted in a lower signal/noise ratio for data acquired on PDMS gels (Fig. S5).

Video S2. Diffusion of A488-Tagged UCHT1 Antibody Fab-Coupled TCRs in rCD2 Contacts Formed on Glass- and 4 kPa PDMS-Supported Lipid Bilayers

### Signaling on PDMS-supported bilayers

**Figure 3:**
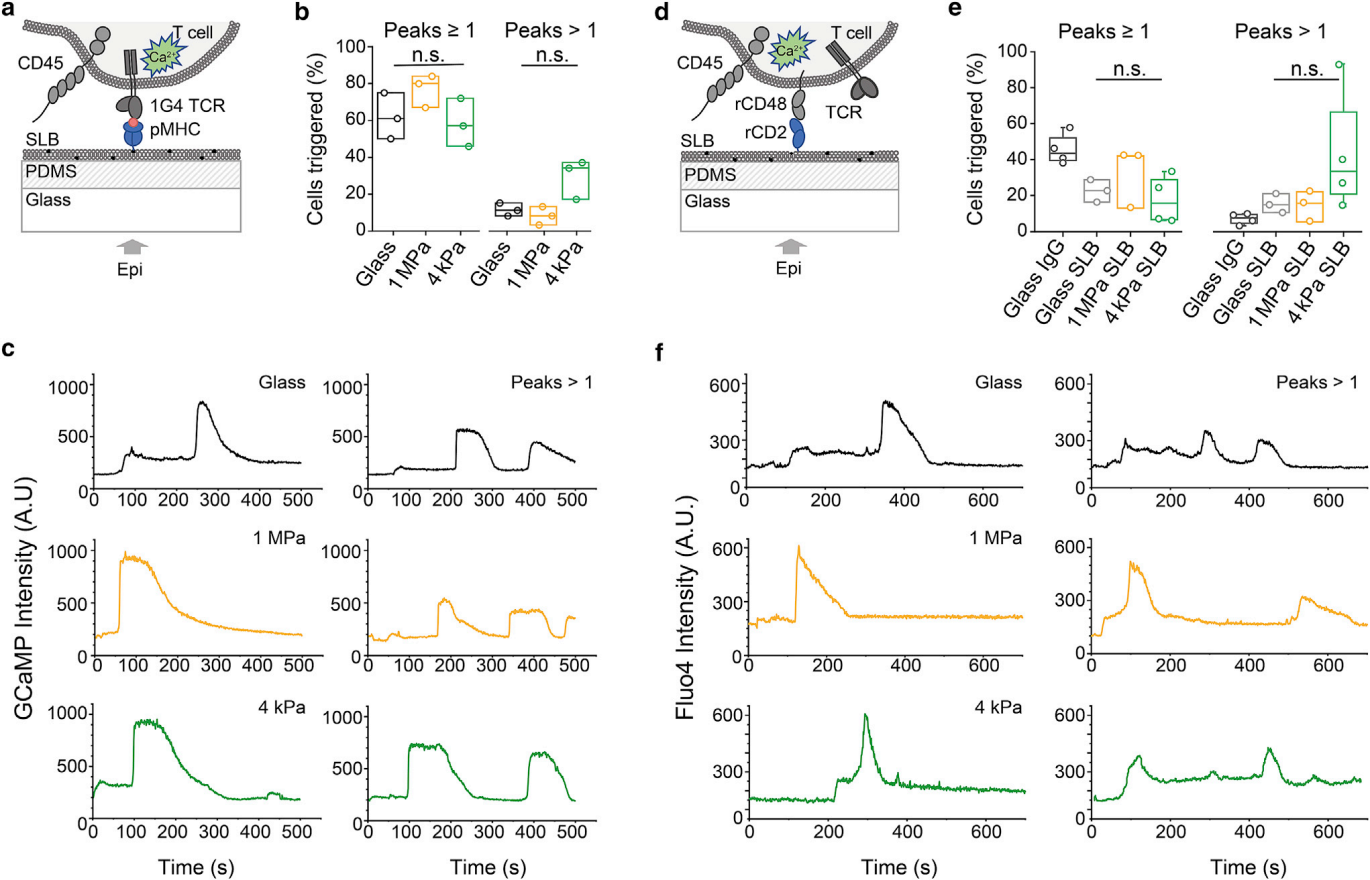
T cell calcium signaling is largely independent of substrate stiffness. (*a*–*c*) Ligand-dependent TCR signaling on pMHC-coated bilayers is shown. (*a*) A schematic representation of the experiment is given. T cells expressing the genetically encoded calcium sensor GCaMP and the 1G4 TCR complex were imaged on pMHC-coated bilayers. (*b*) Boxplots of the fraction of cells exhibiting a calcium response (peaks ≥ 1) and the fraction of triggering cells exhibiting multiple peaks (peaks > 1) are shown for cells interacting with pMHC functionalized glass-supported and 1 MPa and 4 kPa PDMS-supported bilayers. (*c*) Representative calcium traces of cells settling on glass (*black*) and 1 MPa (*orange*) and 4 kPa (*green*) pMHC functionalized SLBs are shown, exhibiting single and multiple peaks (peaks > 1). (*d* and *f*) Ligand-independent TCR signaling is shown. (*d*) A schematic of the experiment is given. Fluo-4 calcium reporter loaded Jurkat T cells expressing signaling-disabled rCD48 were imaged on rCD2-presenting SLBs. (*e*) Boxplots showing fractions of cells exhibiting a calcium response (peaks ≥ 1) and those exhibiting multiple peaks (peaks > 1) on glass coated only with unspecific antibody (bovine immunoglobulin G) and on rCD2-presenting bilayers supported by glass or 1 MPa and 4 kPa PDMS are given. (*f*) Representative calcium traces of cells settling on glass (*black*), 1 MPa (*orange*), and 4 kPa (*green*) rCD2-functionalized SLBs are shown, exhibiting single and multiple peaks (peaks > 1). Each data point represents an independent experiment with n_cells_ > 50. Boxes indicate the 25 and 75% quartile, the horizontal line the mean, and whiskers the 1.5 IQR. All experiments were performed at 37°C. The Mann-Whitney U test was used for statistical analysis. To see this figure in color, go online.

We next sought to determine the influence of substrate stiffness on T cell activation. Whereas previous studies examined the stiffness dependence of activation using late readouts such as IL-2 (4,7,8) or proximal signaling measured as kinase phosphorylation (40), we used calcium signaling responses as an early proximal readout of TCR triggering (Fig. 3 *a*). We used Jurkat T cells transfected with the 1G4 TCR, which binds the NY-ESO 9V peptide presented by the HLA-A2 major histocompatibility complex (pMHC (27)). To detect calcium signaling in different experiments, we used cells expressing the high signal/noise ratio, green-fluorescent-protein-based calcium reporter, GCaMP ((25,26); for cells responding to pMHC), or cells loaded with the calcium indicator dye Fluo-4 (all other experiments).

T cells were first imaged as they contacted bilayers presenting pMHC. Importantly, although substrate resistance has been shown to affect cellular responses (41, 42, 43, 44), we observed that similar large fractions of cells (60–80%) produced calcium signaling responses on glass and on 4 kPa and 1 MPa PDMS-supported bilayers (Fig. 3 *b*). We also observed that a relatively small fraction of the cells exhibited blinking behavior rather than single sustained responses (Fig. 3, *b* and *c*). The fraction of cells exhibiting this blinking behavior was larger on the 4 kPa PDMS-supported bilayers than for 1 MPa PDMS (∼30% vs. ∼10%), although the trend did not reach statistical significance. On bilayers presenting histidine-tagged rCD2 (Fig. 3 *d*), we saw weaker but nontrivial levels of signaling, i.e., in the absence of TCR ligands (Fig. 3, *e* and *f*; Videos S3 and S4), as reported previously (20,45). A time-course analysis showed that even very small contacts with CD2-presenting 4 kPa bilayers were capable of initiating ligand-independent calcium signaling (Fig. S6). These types of responses, which were initially observed by Chang et al. (20) for cells interacting with glass surfaces coated with nonspecific bovine IgG (see also Fig. 3 *e*) and attributed to the effects of local CD45 exclusion, exhibited the same tendency toward increased blinking behavior as substrate stiffness decreased. Finally, similar signaling behavior was observed on PDMS surfaces derivatized with the widely used TCR triggering anti-CD3*ε* antibody, OKT3 (Fig. S7). These findings suggest that regardless of how they are initiated, i.e., by native ligands or antibodies or even in the absence of ligands, the earliest steps in T cell signaling appear to be largely unaffected by substrate resistance.

Video S3. rCD2 Contact Formation and Calcium Signaling on 4 kPa PDMS-Supported Lipid Bilayers at 37°C

Video S4. rCD2 Contact Formation and Calcium Signaling on Glass-Supported Lipid Bilayers at 37°C

## Discussion

Because T cell behavior can be affected by the mechanical properties of surfaces they encounter (4,8,35,40,46), we sought to develop a new model of apposing cell surfaces that replicates their mechanical resistance while preserving the advantages of bilayer technology for studying T cell signaling, i.e., their fluidity and ease of imaging. We have extended the work of others who used PDMS as substrates for culturing T cells. O’Connor et al. used soft (50–100 kPa) PDMS gels to which they adsorbed activating antibodies as a means to expand ex vivo populations of T cells (46). Subsequently, Torres et al. showed that lipid bilayers presenting mobile TCR ligands could be attached to PDMS supports after plasma treatment (7). In this case, the PDMS was used to fabricate microwell arrays for single-cell studies of T cell activation, and their bilayers were presumably relatively stiff. We succeeded in attaching lipid bilayers to very soft PDMS supports of Young’s modulus 4 kPa, with the goal of matching the stiffness of dendritic cells (2–8 kPa (35)), the archetypal antigen-presenting cell. The use of a calcium chloride solution allowed us to avoid plasma treatment, which alters the properties of the substrate (34). Single-molecule tracking and diffusional analysis of labeled lipids in the bilayers confirmed that the surfaces were fluid, and AFM push-through experiments showed that we had single bilayers of the expected height. All measured physical parameters agreed well with the published literature (36,37,47) indicating that, in all other respects, our soft PDMS-supported bilayers matched those prepared on rigid glass supports.

Illustrating their utility, we used the new, soft bilayers to probe the stiffness dependence of surface reorganization and receptor-proximal signaling by T cells by comparing T cell behavior on glass-supported and 4 kPa and 1 MPa PDMS-supported bilayers. TCR diffusion in the absence of ligands was the same for all the bilayers, and we could readily observe the patterns of micrometer-scale spatial reorganization of key cell surface proteins on soft bilayers that had been reported previously on glass surfaces and/or glass-supported bilayers, including the local accumulation of a small adhesion protein, rCD2, and the exclusion from cellular contacts of the large glycocalyx element, CD45 (20,48,49). These findings contrast with those of Cai et al., who did not observe CD45 exclusion from contacts made by T cells interacting with APCs imaged using lattice light-sheet microscopy (23). Whatever the reasons are for this, it seems unlikely that differences in stiffness between cells and glass surfaces are the explanation because, at stiffness levels closely matching those of APCs, we could readily observe CD45 exclusion.

Our measurements of intracellular Ca^2+^ increases, which reports TCR-proximal signaling (50,51), indicated that the earliest stages of T cell activation are largely insensitive to substrate stiffness. There was no reduction in the number of cells responding on 4 kPa vs. 1 MPa PDMS- or glass-supported bilayers and only a modest trend toward an increase in fluctuating Ca^2+^ responses, which might correspond to weaker or incomplete T cell activation (52,53). We obtained similar results for signaling initiated by a mobile pMHC ligand, a directly immobilized antibody, and for triggering induced in the absence of ligands. Previous work showing that substrate stiffness affects T cell activation relied on measurements, for the most part, of late signaling outcomes, i.e., proliferation, cytokine production, and/or the upregulation of surface markers such as CD69 and CD25 (8,35,46). Judokusumo et al., however, examined the effect of substrate stiffness on proximal signaling events, observing only modest (20–30%) reductions in phosphorylated Zap70 and Src-family kinase accumulation on soft (10 kPa) versus more rigid (200 kPa) polyacrylamide gels (40). Interestingly, they also found that on the softer gels, the phosphorylated kinases did not form microclusters, suggesting that microcluster formation requires a degree of substrate resistance, more so than receptor triggering per se. Blumenthal et al. observed much greater effects of hydrogel stiffness on CD4^+^ than on CD8^+^ T cells, also suggesting that whatever mechanism conveys substrate-stiffness effects to the cell lies downstream of the TCR (35). It could be argued that 4- to 10-kPa surfaces are rigid enough to create the tension needed to mechanically trigger the TCR, but even lower levels of rigidity appear to allow TCR triggering. For example, passive contact with ligand-presenting unilamellar vesicles initiates receptor triggering in T and B cells and in mast cells (54). It may also be relevant that TCRs insulated from forces by adhesion molecules on bilayers can be triggered in the absence of ligands (20), as we also observed in this study.

It needs also to be noted that not all studies of the effects of substrate resistance on late-stage signaling outcomes are in full agreement. For example, whereas Saitakis et al. observed a direct correlation between substrate stiffness and the number of proliferating T cells in 72 h cultures on activating antibody-conjugated polyacrylamide gels (8), O’Connor et al. observed fourfold better proliferation of T cells on softer (<100 kPa) rather than harder (>2 MPa) antibody-adsorbed PDMS surfaces (46). For our part, we have to acknowledge that both this and our earlier studies (20,21) were mostly performed using a transformed (Jurkat) cell line. Although we would argue, like others, that used judiciously, such cells can be excellent tools for studying early signaling in lymphocytes (55), differences in membrane composition (56), expression of downstream proteins such as the kinase Itk (57), and observed differences in cytoskeletal rearrangements (58), which all could influence events downstream of TCR triggering, require that our findings are extended to primary cells. We need also to concede that our model system was meant to establish broadly whether phosphatase redistribution is possible on soft surfaces or whether substrate resistance is an essential requirement for receptor triggering, and our bilayers fell well short of representing authentic APC cell surfaces, where lipid and protein diffusion are more constrained (59,60) and many more proteins are present. However, the lipid composition of the bilayer can be easily tuned to mirror the constrained diffusion in APC membranes (61), and more physiological mixes of proteins can be coupled to the SLBs.

Clearly, there is much still to be learned about the mechanobiology of early and late T cell signaling and activation. We find that the new, soft bilayers are stable for at least 4 h, and the work of Judokusumo et al. suggests this might extend to as long as 96 h (40). The stage is therefore now set for systematic studies under conditions closely mimicking physiological cell-cell contact.

## Author Contributions

A.H.L., D.K., and S.J.D. designed the experimental plan. A.H.L., I.B.D., A.K.W., K.Y.C., and J.M. performed experiments and analyzed the data. E.J. and A.M.S. produced the cell lines and purified proteins, and J.H. contributed the calcium analysis code. A.H.L., I.B.D., A.K.W., J.M., K.Y.C., A.M.S., E.J., K.F., S.J.D., and D.K. wrote the article.

## Supporting Material

Document S1. Figs. S1–S7

Document S2. Article plus Supporting Material
