# Supplementary material for: Soft Polydimethylsiloxane-Supported Lipid Bilayers for Studying T Cell Interactions
Source: Biophys J. 2020 Nov 26;120(1):35–45. doi: 10.1016/j.bpj.2020.11.021 (PMC7820804; doi:10.1016/j.bpj.2020.11.021)
Supplement: Document S1. Figs. S1–S7 [file mmc1.pdf]

Biophysical Journal, Volume *120*

Supplemental Information

Soft Polydimethylsiloxane-Supported Lipid Bilayers for Studying T Cell Interactions

Anna H. Lippert, Ivan B. Dimov, Alexander K. Winkel, Jane Humphrey, James McColl, Kevin Y. Chen, Ana M. Santos, Edward Jenkins, Kristian Franze, Simon J. Davis, and David Klenerman

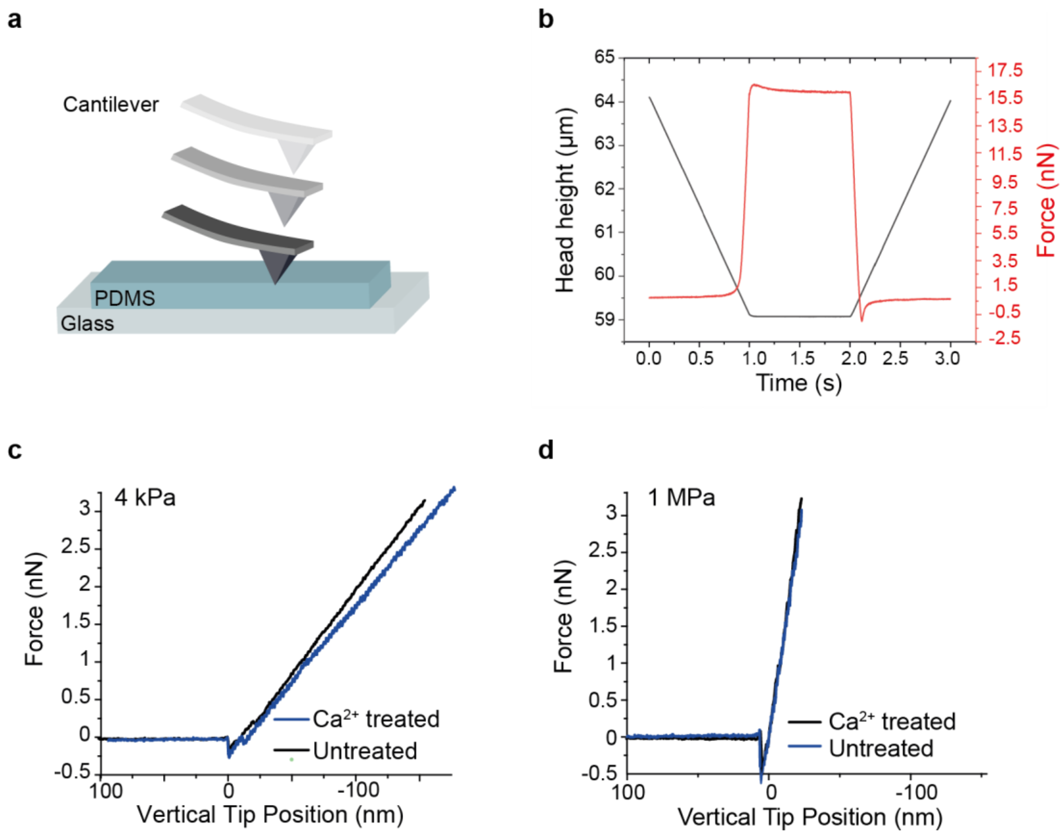

Suppl. Fig. 1: PDMS characterisation using AFM. (**a**) Schematic of the experiment. (**b**) Creep response curve of 4 kPa gel, recording the force on the cantilever (red) at constant cantilever head height (black). (**c, d**) Force response of indentation experiments plotting force over vertical tip positions comparing Ca^{2+} treated (blue) and untreated (black) 4 kPa (**c**) and 1 MPa (**d**) PDMS gels.

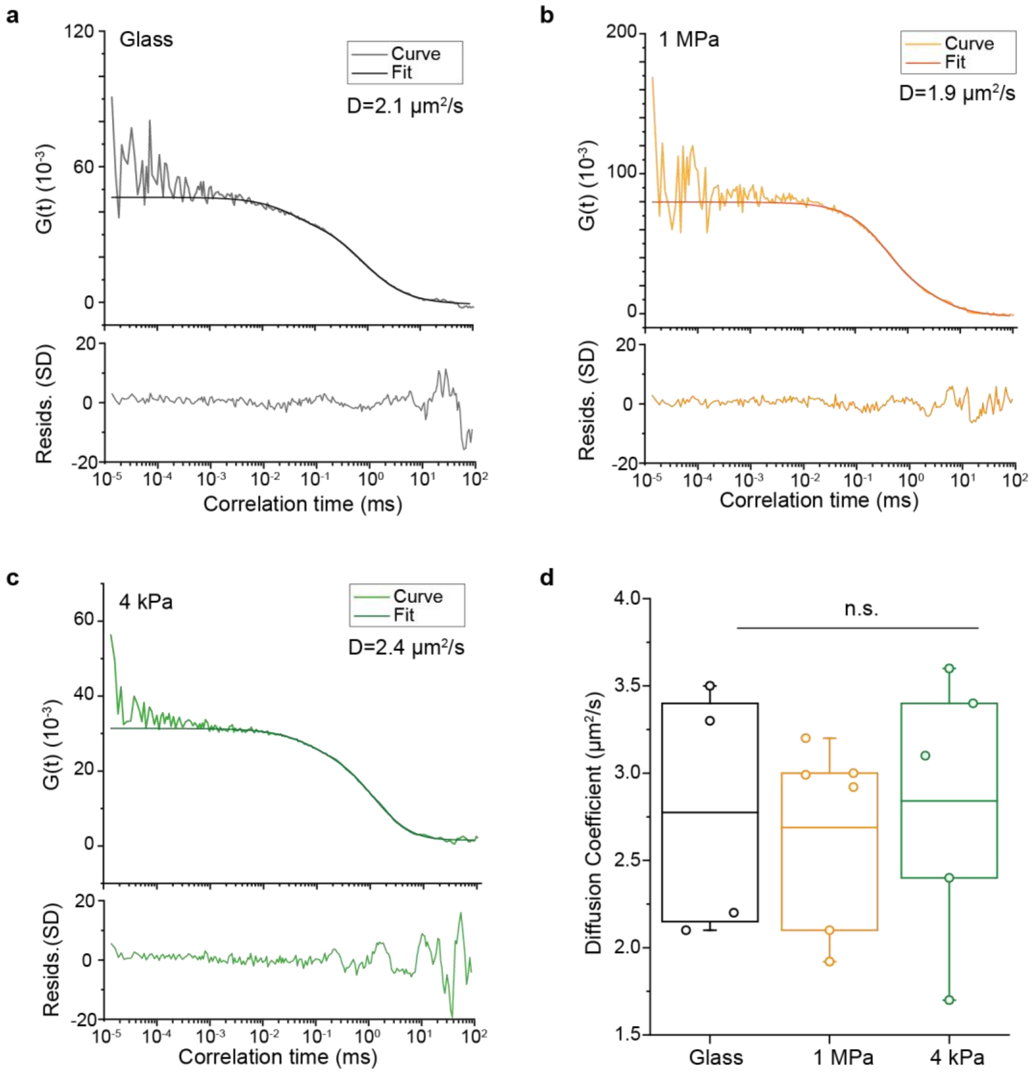

Suppl. Fig. 2: FCS measurements of glass- (**a**), 1MPa (**b**) and 4 kPa (**c**) PDMS-supported bilayers. Shown are representative autocorrelation curves G(t) and their respective fits as well as a boxplot comparing diffusion coefficients (**d**). Each data point represents the diffusion coefficient obtained from one bilayer at RT. Boxes indicate the 25% and 75% quartile, the horizontal line the mean, and whiskers the 1.5 IQR. A Mann-Whitney-U test was performed to test for significant differences.

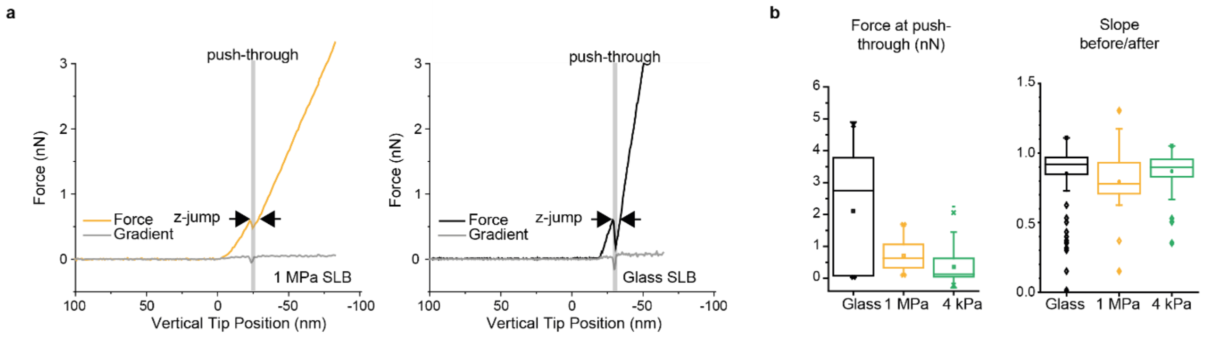

Suppl. Fig. 3: AFM-based characterisation of glass-supported and 1 MPa and 4 kPa PDMS-supported lipid bilayers. (**a**) Representative force trace of a cantilever pushing through a 1 MPa (force: orange) and glass supported bilayer (force: black) and gradient of the force (grey). The push-through event is indicated as a "z-jump" (grey area with indicating arrows). (**b**) Boxplots comparing push-through force (left) and slope before/after the push-through (right) (number of force curves analysed: 52 (4 kPa PDMS), 30 (1 MPa PDMS), 31 (glass) from 3 independent experiments). Boxes indicate the 25% and 75% quartile, the square is the mean, the horizontal line the median, the diamonds the outliers and whiskers the 1.5 IQR.

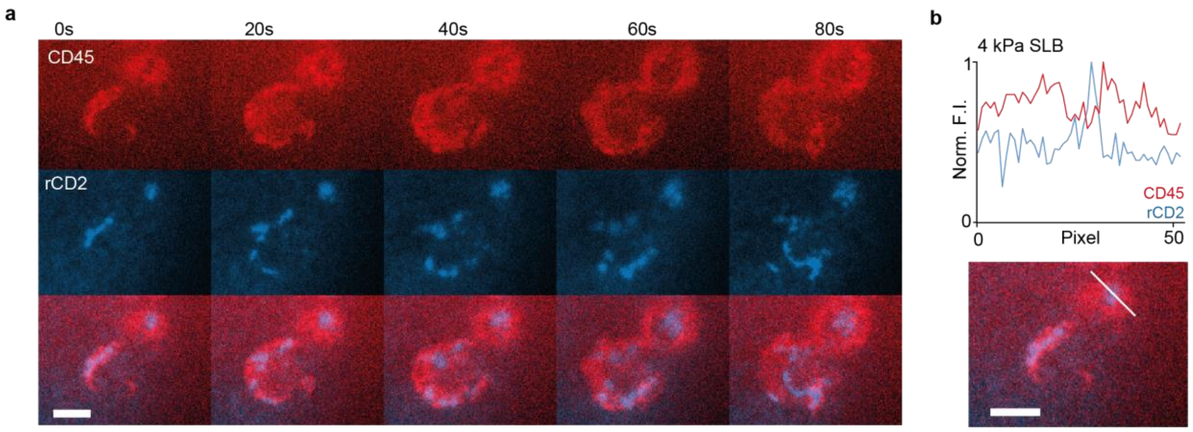

Suppl. Fig. 4: Small contacts exclude CD45. (**a**) Representative false-colour two-colour time-lapse TIRF imaging of a T cell recruiting rCD2-Alexa647 (blue) into contacts, alongside exclusion of CD45 labelled with Gap8.3 anti-CD45 antibody Fab tagged with Alexa 488 (red). (**b**) Line profile of CD45 and rCD2 fluorescence at the 0s timepoint. Scale bars 1μm.

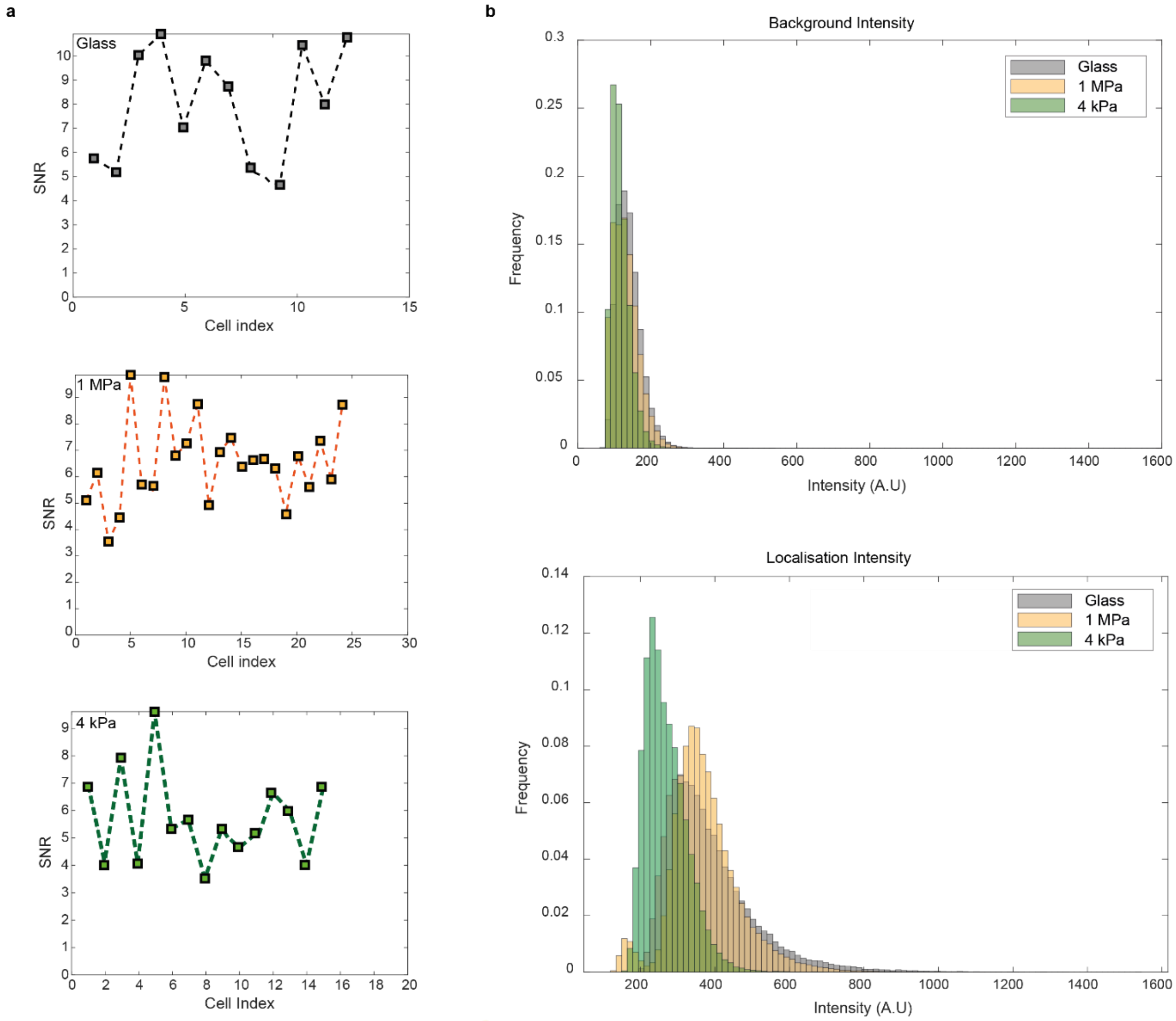

Suppl. Fig. 5: Signal-to-noise (SNR) ratios of UCHT-1 Alexa488 diffusion data shown as SNR cell by cell (**a**) and as histograms of overall background (**b**, top) and localisation intensities (**b**, bottom).

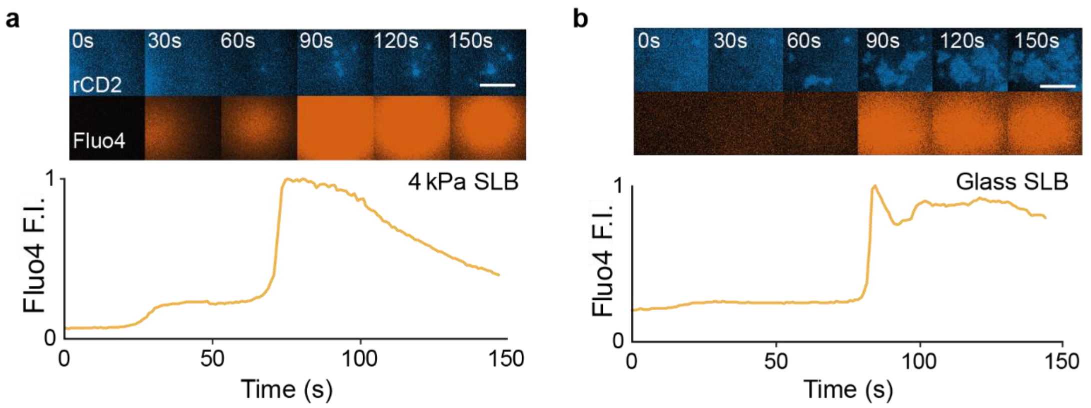

Suppl. Fig. 6: Small rCD2 contacts exclude CD45 and produce calcium responses on 4 kPa PDMS-supported bilayers. Jurkat cells expressing rCD48 were loaded with Fluo-4 and allowed to settle onto Alexa647-tagged rCD2-presenting 4 kPa PDMS (**a**) and glass (**b**) supported bilayers. Two colour illumination with a 488 laser set to epi illumination and a 647 laser set to TIRF illumination allowed simultaneous detection of rCD2 accumulation and calcium signaling. The Fluo4 signal, as raw images and traces (orange) are shown under the time-lapse TIRF images. Scale bars 5 µm.

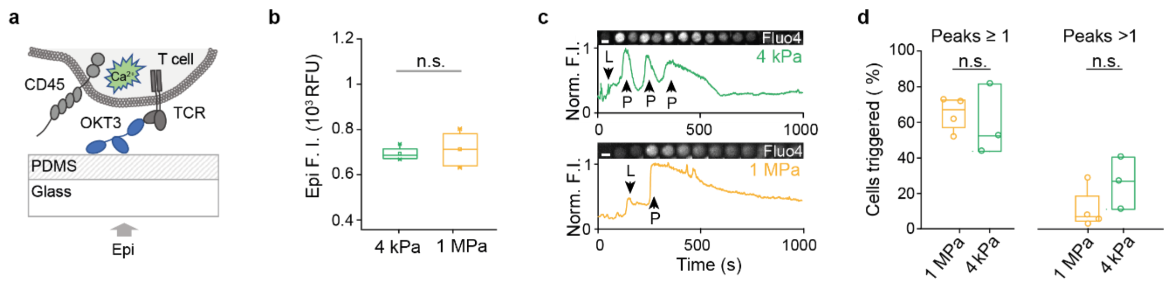

Suppl. Fig. 7: Calcium responses of T cells on PDMS coated with activating OKT3 anti-CD3ε antibody. (**a**) Schematic of the experiment. T cells were preloaded with Fluo-4 prior to imaging their calcium responses on OKT3-coated PDMS. (**b**) PDMS gel stiffness did not influence antibody loading at the concentration used (10 µg/ml). Gels were incubated with fluorescently labelled antibody overnight, washed and the fluorescence intensity measured using epi-illumination. A two-sided t-test was used for statistical analysis. (**c**) Example calcium signaling traces for cells contacting OKT3 antibody-coated 4 kPa and 1 MPa PDMS gels; L, cell landing, P, calcium peaks. Fluo4 fluorescence is shown above the trace. Scale bar 10 µm. (**d**) Fraction of cells exhibiting a calcium response (peak ≥ 1) and fraction of responding cells that displayed multiple peaks (peaks > 1). Each data point in the boxplot represents a single experiment with $n_{cells}>50$. All experiments were performed at 37°C. The Mann-Whitney U test was used for statistical analysis.
